# Use of an Online Forum for Relatives of People With Psychosis and Bipolar Disorder: Mixed Methods Study

**DOI:** 10.2196/35837

**Published:** 2022-10-20

**Authors:** Steven Jones, Dimitrinka Atanasova, Susanna Dodd, Susan Flowers, Anna Rosala-Hallas, Heather Robinson, Elena Semino, Fiona Lobban

**Affiliations:** 1 Spectrum Centre for Mental Health Research Division of Health Research Lancaster University Lancaster United Kingdom; 2 DisTex - Discourse and Text Research Group Lancaster University Lancaster United Kingdom; 3 Department of Biostatistics Clinical Trials Research Centre University of Liverpool Liverpool United Kingdom; 4 Department of Linguistics and English Language Lancaster University Lancaster United Kingdom

**Keywords:** psychosis, bipolar disorder, relative, carer, mental health, forum, online, digital health, Relatives Education and Coping Toolkit, REACT, trial

## Abstract

**Background:**

Relatives of people with psychosis or bipolar disorder experience high levels of distress but are typically not offered the support they need. Online peer forums may offer a solution, but knowledge about who uses them, how, and why is limited. This study reported on online forum use during the Relatives Education and Coping Toolkit (REACT) trial.

**Objective:**

We aimed to report who used the forum and why; how sociodemographic factors are associated with participation; the relationship among frequency, type of use, and outcomes; and how the forum was used.

**Methods:**

The relationships between key sociodemographic characteristics, levels of forum use, and distress were statistically analyzed. We used thematic and semantic analyses to understand the reasons for relatives joining the forum and the key topics initiated by them. We also used the University Centre for Computer Corpus Research on Language Semantic Analysis System to compare how relatives and REACT supporters (moderators) used the forum.

**Results:**

A total of 348 participants with full forum use data from REACT were included in this study. The forum was accessed by 59.4% (207/348) of the relatives across the entire age range, with no significant associations between sociodemographic factors and forum participation, or between level or type of use and relatives’ distress levels. Relatives joined the forum primarily to find people in similar circumstances, express concerns, and talk about stressful events. Relatives were most concerned about recent events, negative emotions linked to caring, experiences of conflict or threat, and concerns about suicide. These posts underscored both the challenges the relatives were facing and the fact that they felt safe sharing them in this context.

**Conclusions:**

Although only a proportion of REACT participants engaged actively with its forum, they were widely distributed across age and other sociodemographic groupings. Relatives used the forum for information, support, and guidance and to offer detailed information about their experiences. The topics raised highlighted the burden carried by relatives and the potential value of easy-access, moderated, peer-supported forums in helping relatives to manage the challenges they faced.

## Introduction

### Background

Psychosis and bipolar disorder (BD) are severe mental health problems affecting 2% to 4% of the global population, respectively [1,2], with a cost of £9.2 billion (US $10.1 billion) per year to the English economy [3]. Relatives of individuals with these conditions deliver vital but unpaid care [4]. However, this caring role often comes at a huge cost to the relatives themselves in terms of burden and distress [5,6].

There is increasing awareness of the need to support relatives of people with mental health problems [7]. The National Institute for Health and Care Excellence [8] recommends that relatives receive education, information, and support [9]. With support, relatives have better health outcomes [10].

Despite this, most relatives receive little support [11-13]. Furthermore, most evidence is for face-to-face interventions, which have not been widely adopted. This reflects a lack of provision of family interventions across the United Kingdom as well as some relatives not taking up such approaches when offered. The Royal College of Psychiatrists Report of the Early Intervention in Psychosis Audit, for instance, indicated that of more than 1901 families in early intervention services, only 31% were offered family intervention, of whom 38% took up this offer [14]. A crucial question is how support for relatives can be delivered accessibly and cost-effectively at scale. Web-based interventions have been established for several mental health conditions, including depression and anxiety [15,16], with increasing evidence for the benefits for people with severe mental health problems [17] and their relatives [12,18]. Online forums offer an accessible space where users can connect anonymously [19], with growing evidence of the benefits of forum engagement [20]. However, the use of forums by relatives has been largely ignored, with some exceptions [21-24], especially for relatives of people with mental health difficulties.

### Objectives

This paper aimed to report on forum use from a large national UK digital mental health trial (Relatives Education and Coping Toolkit [REACT]) [24-26]. The REACT trial found that the intervention was inexpensive and acceptable, and was a safe method of delivering support for relatives of people with psychosis and BD. Both the REACT intervention and access to a digital resource directory were associated with significant increases in carer well-being and reduction in distress; however, there was no difference between the 2 trial arms in these outcomes at either 12 or 24 weeks of follow-up. Of the 800 participants in the REACT trial, 399 (49.8%) were in the active intervention arm, with access to a peer-supported moderated forum. Here, we report on their patterns of REACT forum use during the trial. Specifically, we aimed to explore (1) who used the forum and why; (2) how sociodemographic characteristics are associated with participation, taking into consideration previous research on patterns of use of digital resources linked to age, sex, education, and employment or income [27]; (3) the relationship among frequency, type of use, and outcomes; and (4) how the forum was used.

## Methods

### The REACT Forum and Trial

**Figure 1:**
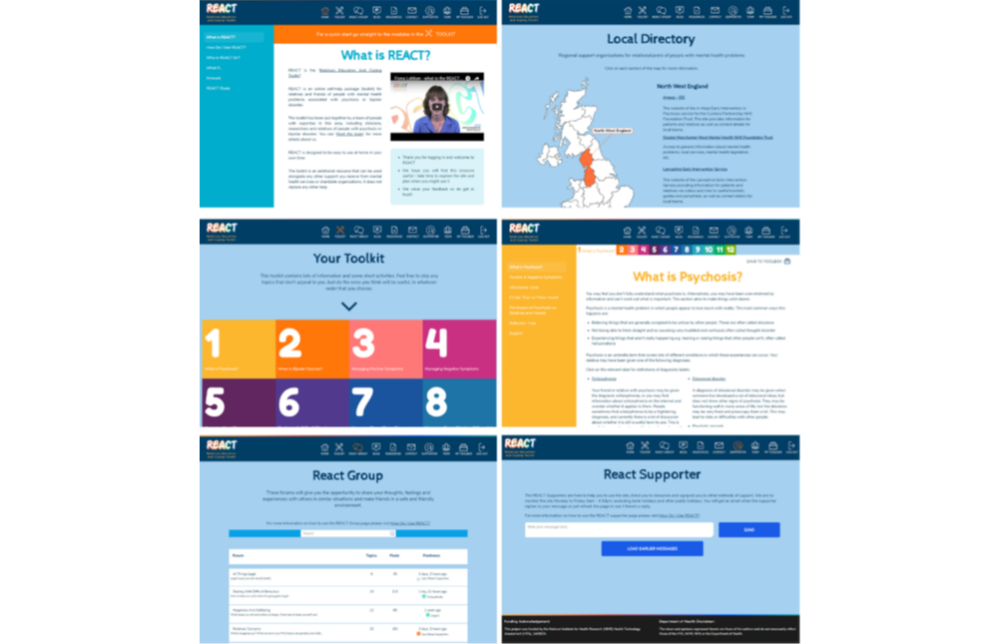
Screenshots of Relatives Education and Coping Toolkit (REACT).

REACT was originally developed as a printed toolkit or web page and reduces relatives’ distress [26]. To increase access and flexibility, REACT was adapted into an internet-based digital intervention [25] built in WordPress (Automattic Inc.) with a number of plug-ins. These included bbPress (Automattic Inc.) to run the REACT group forum. The content of the toolkit was informed by family intervention models for people with psychosis [11]. The key components of the toolkit were as follows: 12 information modules, a comprehensive resource directory, a group forum, and a confidential direct messaging service. A *meet the team* page ensured that relatives were fully informed about who was delivering the content of the site. *Mytoolbox* offered users a confidential space to save links to any information they might keep. A blog page offered a flexible space for additional communication with site users, edited by the REACT supporters. The screenshots in Figure 1 show the look and feel of the REACT website.

REACT users were offered support through confidential direct messaging with REACT supporters and peer support through a moderated online forum. The REACT supporters were available on the site from Monday to Friday, from 9 AM to 4:30 PM, excluding bank holidays and university holiday closures. Their key role was to provide emotional support and to guide relatives to relevant parts of the toolkit or other resources, as appropriate.

### Training REACT Supporters From the REACT Trial

The REACT supporters had experience of caring for someone with BD or psychosis, but were otherwise not required to have any other educational or clinical experience. REACT supporters were trained to moderate the online REACT forum and to respond to direct confidential messages. The training was provided by the clinical supervisor (SJ) and the chief investigator (FL) before the launch of REACT and focused on providing empathetic support and guiding relatives to use the toolkit in the best way to help them with their concerns. The supporters were not formally clinically trained, as their focus was on providing support and specifically not on providing advice. This preparatory work included group discussions on the nature of the work and the distinctions between support and advice. REACT supporters also reviewed vignettes of possible forum posts to gain practice in response options in advance of the REACT site going live. Potential responses were reviewed with SJ. REACT supporters then received regular supervision from SJ, covering issues raised on the forum and any risk concerns, as well as supporter well-being and ensuring forum coverage during periods of leave. These sessions occurred 1.8 times per month on 1-2 times per month across the duration of the trial. SJ was also available for ad hoc meetings, when required. The IT support lead (Andrew Walker) provided technical training to ensure that the supporters were very familiar with the whole of the REACT site and with how to access the automated emails informing them about posts. The REACT supporters were also provided with, and helped develop, several written documents: a training document regarding supporter roles and the site; a REACT Supporter Manual that included examples of posts and risk emails; a thorough Risk Protocol and Matrix that outlined what to do in the event of risk being identified on the site; and several documents regarding the use of the site, for example, how to search for a forum post and how to hide inappropriate content.

REACT supporters were required to check the forum at least three times a day, from Monday to Friday. They identified dangerous behaviors or concerns, categorizing them as low or high risk. Low risk was defined as no indication of immediate or serious threat of severe harm or risk to life but the presence of either clear evidence of high levels of distress or concerns for risk of harm or abuse toward participants or others (safeguarding risks). Where high distress was identified, REACT supporters responded with a standardized email. Where a safeguarding issue was identified, REACT supporters consulted their clinical supervisor and National Health Service trust safeguarding team. High risk was defined as the presence of clear evidence of immediate and serious risk to life or child welfare. If an immediate risk was identified, the REACT supporters called the police or social services, depending on the risk. Risk was identified based on participants’ post content rather than on their post frequency. REACT supporters also monitored the forum for inappropriate posts and could hide them if necessary. All risks and inappropriate posts were discussed during clinical supervision or at an earlier meeting if needed.

REACT also contained an extensive resource directory. The REACT trial was a web-based, 2-arm, pragmatic randomized controlled trial comparing the REACT intervention to only providing access to the resource directory. Outcomes were assessed at baseline, the 12-week follow-up, and the 24-week follow-up. The primary outcome was relatives’ distress, assessed using the General Health Questionnaire (GHQ)-28 [28]. Participants were recruited to the REACT trial from April 22, 2016, to September 30, 2017. A range of web-based (Facebook, Twitter, and charity websites) and offline recruitment strategies (clinical services and third sector providers) were used, all directing potential participants to the study home page.

### Participants

Participants in this study comprised 348 individuals randomized into the REACT intervention arm. From the original 399 participants allocated to REACT, 51 (12.8%) were excluded because their complete web use data were not recorded.

The inclusion criteria were (more details are presented in the trial paper by Lobban et al [24]) the following:

- Aged ≥16 years
- Living in the United Kingdom
- Relative or close friend of someone with psychosis or BD
- Currently experiencing distress
- Currently seeking help (self-identified)
- Having access to the internet
- Sufficient English fluency to comprehend the intervention and forum content

Participants were identified as “currently experiencing distress” because of their relative or close friend, by their selecting “rather more than usual” or “much more than usual” on the GHQ-28 item “Have you been feeling nervous and strung up all the time?” This was included to avoid a floor effect on levels of distress at baseline, and this item was used because it correlated most highly with the GHQ-28 score in the REACT feasibility trial [26]. An age cutoff of ≥16 years was used as this is the legal age of consent, and the REACT intervention aimed to be inclusive of all carers of people with psychosis or bipolar; many carers were aged 16 to 18 years.

### Analysis

#### Overview

Participants’ forum activity was recorded from randomization until the date of their primary outcome assessment (GHQ-28 score at 24 weeks) or, if the assessment was not completed, the date on which it would have taken place. Participants were classified based on their use levels as nonusers (did not access forum at all), observers (accessed the forum but did not post), or users (accessed and posted on the forum at least once). Analyses were conducted using Stata (StataCorp; version 14). A Cronbach α level of *P*<.05 was used as a general indicator of statistical significance. A complete case analysis approach was adopted. Use levels of the forum were identified based on participant IDs. As there were a number of exploratory analyses, all results have been interpreted cautiously.

#### Who Used the Forum and for What Reasons

To outline who used the forum, descriptive statistics were calculated based on the level of use and different demographic factors (age, gender, highest education level, and employment status).

To explore why relatives started using the forum, 2 coauthors (DA and ES) classified the functions of relatives’ first posts by adapting the Rohr [29] coding scheme. The Rohr coding scheme is based on the concept of “discursive moves” defined as the kinds of contributions that entries make to the ongoing interchange, in turn based on the Locher [30] and Morrow [31] catalog of discursive moves. All functions were retained from the Rohr coding scheme except functions associated with the moderators’ posts (eg, “official welcome,” described as users being welcomed to the forum by a moderator). We inductively added the category “other” for posts discussing technical aspects of the forum’s use. Initially, a small random subsample of first posts was examined to ensure that they would be categorized into the same theme by the 2 coauthors. A standardized procedure for coding agreement such as interrater reliability was not adopted because a reflexive approach to thematic analysis was used. As Braun and Clarke [32] write, “this approach fully embraces qualitative research values and the subjective skills the researcher brings to the process—a research team is not required for quality.”

The analysis focused on the messages written by 19.2% (67/348) of the relatives, which is a sample of participants who had complete web use data available. One first post could be coded for more than one function.

#### Sociodemographic Factors Associated With Participation

Associations between sociodemographic factors (age, gender, education level, and employment status) and forum use levels were compared using Fisher exact tests, split according to no active participation (no posts), low active participation (up to five forum posts), and high active participation (>5 forum posts). A total of 5 forum posts were chosen as a cutoff based on a consensus team decision that 5 posts is the minimum number to be actively engaged with the forum; the 5-post cutoff identified the top one-third of the posters.

#### Relationship Among Frequency, Type of Use, and Outcomes

We calculated the mean GHQ-28 scores (the primary outcome of the REACT trial) at each time point (baseline, 12 weeks, and 24 weeks) and for each forum use level (nonuser, observer, and user). Spearman correlation coefficients were used to assess the relationship between the number of forum posts and GHQ-28 scores.

#### How Was the Forum Used?

To explore how relatives and REACT supporters used the forum differently, 2 data sets were created, consisting of the following categories:

- Topics initiated by relatives (“User Topics”): 33,201 words
- Topics initiated by supporters (“Supporter Topics”): 335,819 words

To explore potential differences between the 2 data sets, the University Centre for Computer Corpus Research on Language Semantic Analysis System (USAS) was used in the web-based software Wmatrix [33].

USAS is a tagging program that automatically assigns a semantic category label (or semantic tag) to every word or phrase in a linguistic data set, or “corpus.” The category scheme consists of 21 general semantic domains (eg, “Emotion”) and 232 more specific subdomains (eg, “Sad” as a subdomain of “Emotion”). Unlike other types of content analysis systems such as Linguistic Inquiry and Word Count [34], the USAS tagger takes into account the meaning of a word or phrase in context to assign an appropriate tag. A central aspect of this contextual disambiguation is the tagger’s ability to assign single tags to phrases or multiword expressions, including phrasal verbs (eg, “look after”) and proper names (eg, “Milton Keynes”). The tool has been shown to have a level of accuracy of approximately 91% [35].

We used the web-based concordancer, Wmatrix, which applies the USAS semantic tagger to any text loaded into the system, to compare topics initiated by relatives with topics initiated by REACT supporters in terms of the relative frequencies of the 232 specific semantic domains to establish which semantic domains are “key” or “overused” in the former compared with the latter, according to the following 2 statistical measures:

- LogRatio: a measure of effect size, that is, the binary log of the ratio of relative frequencies in the 2 data sets [36].
- Log Likelihood: a measure of statistical significance that is sensitive to the size of the evidence that a difference exists [29].

We set a minimum LogRatio score of 0.5, meaning that the relevant semantic domain is 50% more frequent in the target corpus (relatives) than in the reference corpus (supporters) and a minimum Log Likelihood threshold of 6.63, providing a confidence measure equivalent to *P*=.01 [37].

### Ethics Approval

All procedures contributing to this work comply with the ethical standards of the relevant national and institutional committees on human experimentation and with the Helsinki Declaration of 1975, as revised in 2008. All participants provided written informed consent, and all procedures were approved by the Lancaster National Research Ethics Service Committee (15/NW/0732).

## Results

### Who Used the Forum and for What Reasons

Active forum use was distributed across all age bands of the trial, except for those aged ≥70 years. For this age group, participants were either nonusers or only observers (Table 1). Most forum participants were considered observers. The users were aged 40 to 69 years. Overall, more women than men participated in the REACT forum, with a higher percentage of women in the observer and user groups. There were few differences in education, except for a suggestion that users were more likely to have engaged with higher education. No meaningful differences were observed in terms of employment, which was similarly distributed across no paid employment, part-time employment, and full-time employment for all 3 groups.

**Table 1:** Demographic characteristics of forum users (N=348).

Characteristic			Nonuser (never logged in; N=141), n (%)		Observer (no posts; N=140), n (%)		User (at least one post; N=67), n (%)		Total
**Age (years)**									
	<30	19 (13)		13 (9)		3 (4)		35	
	30-39	18 (13)		15 (11)		12 (18)		45	
	40-49	39 (28)		30 (21)		13 (19)		82	
	50-59	31 (22)		45 (32)		21 (31)		97	
	60-69	25 (18)		31 (22)		18 (27)		74	
	≥70	9 (6)		6 (4)		0 (0)		15	
**Sex**									
	Female	99 (70)		114 (81)		58 (87)		271	
	Male	42 (30)		26 (19)		9 (13)		77	
**Highest education level**									
	School level	25 (18)		25 (18)		7 (10)		57	
	Further (UK college level)	41 (29)		39 (28)		18 (27)		98	
	Higher (UK university level)	75 (53)		76 (54)		42 (63)		193	
**Employment status**									
	None or unpaid	54 (38)		59 (42)		25 (37)		138	
	Part-time	32 (23)		32 (23)		16 (24)		80	
	Full-time	55 (39)		49 (35)		26 (39)		130	

The total word count of the first 67 posts was 14,070. Posts varied between 25 and 866 words, with a mean of 210 words. Posts were manually coded for the following functions to capture patterns in content and interactional characteristics.

- *greeting*: a formal opening move (eg, “Hello”)
- *meta-comment*: a comment on the experience of using the forum (eg, “New here so just finding my way about”)
- *background information*: provide information about oneself or the issue at hand (eg, “My wife suffers some severe symptoms, has many triggers”)
- *request for advice or information or support*: asking for guidance (eg, “So is there any advice or techniques anyone can suggest”)
- *provision of advice or information or support*: providing guidance (eg, “So if you have a crisis team, I would recommend contacting them”)
- *thank*: thank in anticipation of the advice or information or support, or for writing or reading (eg, “Thank you,” “Thanks for sharing this”)
- *well-wishing*: wish someone well (eg, “All the very best for the future”)
- *farewell*: a formal closing move (eg, “Regards”)

A total of 22 (32.8%) posts began with a greeting; 21 (31.3%) made a meta-comment about using the forum; 64 (95.5%) provided background information about the relative or the issue that prompted them to post on the forum; 14 (20.9%) included a request for advice, information, and support; 13 (19.4%) provided advice about possible courses of action; 11 (16.4%) expressed gratitude to others for advice or simply for writing and reading posts; 3 (4.5%) included well-wishes; and 1 (1.5%) ended with a formal farewell.

Most posts (53/67, 79.1%) provided information about the posters’ circumstances, without requests for advice or information. This suggests that the forum was primarily used to find people in similar circumstances, express concerns, and talk about stressful events. Some contributors made this explicit, with comments such as “Hello, I am so happy I have found this link! All your messages are deeply resonating in me.”

### Are Sociodemographic Factors Associated With Participation?

We looked at the distribution of the total number of forum posts across all participants except for a single outlier (who posted in the forum 205 times) to show the distribution of lower values more clearly. This revealed a significant negative skew, with 81% (281/348) of the sample not posting at all and 93% (325/348) of the sample posting ≤5 times. The average number of posts, with outliers excluded, was 6.8 (SD 9.3).

Participants were classified as having “highly participated” if they were in the top one-third of active forum users, that is, those who posted >5 times in the forum (Table 2). There were no statistically significant relationships indicated among age, gender, education or employment, and level of forum use. Numerically there was an indication that the high-use group had a preponderance of participants aged 50 to 59 years, but the association between high or low use was not significant.

**Table 2:** Demographic characteristics of relatives according to amount of forum use (N=348).

Characteristic			No active participation (no posts; N=281), n (%)		Low active participation (1-5 posts; N=44), n (%)		High active participation (>5 posts; N=23), n (%)		Total		Fisher exact test, *P* value	
**Age (years)**												.27
	<30	32 (11)		3 (7)		0 (0)		35				
	30-39	33 (12)		8 (18)		4 (17)		45				
	40-49	69 (25)		9 (20)		4 (17)		82				
	50-59	76 (27)		11 (25)		10 (43)		97				
	60-69	56 (20)		13 (30)		5 (22)		74				
	≥70	15 (5)		0 (0)		0 (0)		15				
**Sex**												.18
	Female	213 (76)		38 (86)		20 (87)		271				
	Male	68 (24)		6 (14)		3 (13)		77				
**Highest education level**												.64
	School level	50 (18)		4 (9)		3 (13)		57				
	Further (UK college level)	80 (28)		12 (27)		6 (26)		98				
	Higher (UK university level)	151 (54)		28 (64)		14 (61)		193				
**Employment status**												.94
	None or unpaid	113 (40)		15 (34)		10 (43)		138				
	Part-time	64 (23)		11 (25)		5 (22)		80				
	Full-time	104 (37)		18 (41)		8 (35)		130				

### Relationships Among Frequency, Type of Use, and Outcomes

The primary outcome for the REACT trial was the GHQ-28 scores. Therefore, we explored the relationship between this and forum use based on the nonuser, observer, and user categories. The mean levels of GHQ scores at each time point are very similar for each use group (Table 3).

Spearman correlations were calculated for those who used the forum to explore any relationships between forum use and GHQ-28 scores. As Table 4 indicates, all these correlations were small and nonsignificant.

**Table 3:** General Health Questionnaire-28 (GHQ-28) scores of relatives by time and level of forum use.

Values	GHQ-28 at baseline				GHQ-28 at 12 weeks				GHQ-28 at 24 weeks		
	Nonuser (n=141)	Observer (n=140)	User (n=67)	Nonuser (n=77)		Observer (n=108)	User (n=60)	Nonuser (n=83)		Observer (n=110)	User (n=59)
Value, mean (SD)	40.9 (15.6)	39.8 (13.4)	41.2 (16.0)	29.9 (16.3)		31.8 (17.0)	29.9 (12.3)	30.2 (17.8)		30.7 (16.0)	28.7 (16.2)
Value, median (IQR)	39 (29-51)	39 (29-49)	38 (29-53)	26 (17-42)		29 (19.5-39)	28 (22-35.5)	26 (16-43)		28 (18-41)	26 (17-37)
Value, range	5-83	18-83	17-76	4-73		5-80	3-63	2-76		6-78	5-79
Missing, n (%)	0 (0)	0 (0)	0 (0)	64 (45)		32 (23)	7 (10)	58 (41)		30 (21)	8 (12)

**Table 4:** Correlations between forum use and General Health Questionnaire-28 (GHQ-28) scores at each assessment point.

Assessment point for GHQ-28 score	Spearman correlation coefficient (*P* value) for forum users only	Spearman correlation coefficient (*P* value) for forum users only (outlier removed)	Spearman correlation coefficient (*P* value) for all REACT^a^ participants (outlier removed)
Baseline	−0.003 (.98)	0.005 (.96)	0.006 (.91)
12 weeks	−0.113 (.38)	−0.071 (.59)	0.005 (.94)
24 weeks	−0.091 (.49)	−0.054 (.68)	−0.034 (.58)

^a^REACT: Relatives Education and Coping Toolkit.

### How Was the Forum Used?

Only REACT supporters could start a new thread (eg, include “happiness and wellbeing,” “treatment services,” “all things legal,” and “dealing with difficult behaviour”), but relatives could start a new topic within an existing forum, which is something they did 131 times. These relative-initiated topics (and their descriptions) totaled 33,201 words (User Topics), in contrast with supporter-initiated topics, which totaled 335,819 words (Supporter Topics).

Table 5 lists the 21 semantic domains statistically overused in User Topics than in Supporter Topics. The rightmost column gives examples of the words included under each semantic domain.

The 21 overused domains include the following major themes:

- Time, including beginnings, endings, age, and recency
- Negative emotions, including fear, depression, and anxiety
- Conflict and abuse, including anger, threats, and abuse
- Illness and hospitalization, including psychosis and discharge
- Death and suicide

Overall, this suggests that users typically initiated topics to talk about particularly acute problems relating to their relative’s condition and, therefore, their own situation. These problems sometimes involve actual, feared, or threatened violence or suicide, and have often worsened before the decision to write on the forum. Some examples are given in Table 6, which indicate both the extent of the challenges experienced by these relatives and their frankness about them on the forum.

**Table 5:** Semantic domains used more by relatives than supporters.

Code	Raw frequency Corpus 1	Relative frequency Corpus 1	Raw frequency Corpus 2	Relative frequency Corpus 2	LL^a^	LogRatio	Label	Example words
X7-	17	0.05	20	0.02	10.79	1.63	Unwanted	*Rubbish* ^b^ *, rejected*
A13.4	63	0.20	90	0.08	29.58	1.35	Degree: Approximators	*About, almost*
T3	48	0.15	70	0.06	21.73	1.32	Time: Old, new and young; age	*Age, one, aged*
T1.1	66	0.21	102	0.09	26.80	1.23	Time: General	*Appointment or appointments*
M3	54	0.17	86	0.08	20.66	1.19	Vehicles and transport on land	*Car, drive*
A1.7-	35	0.11	57	0.05	12.79	1.16	No constraint	*Discharge or dishcarged, escape*
T3-	27	0.09	47	0.04	8.51	1.06	Time: New and young	*Recently*
E5-	59	0.19	103	0.09	18.48	1.06	Fear or shock	*Scared, fear, terrified*
H4	65	0.21	118	0.10	18.53	1.00	Residence	*Home, live*
E4.1-	101	0.32	192	0.17	25.55	0.93	Sad	*Depression, depressed*
L2	28	0.09	54	0.05	6.81	0.91	Living creatures: animals, birds, etc	*Dog, cat or cats*
L1-	31	0.10	60	0.05	7.47	0.91	Dead	*Suicide, death, died, suicidal*
T1.1.1	150	0.47	311	0.27	29.45	0.81	Time: Past	*Last year, yesterday*
T3-	54	0.17	121	0.11	8.11	0.70	Time: New and young	*New*
E3-	91	0.29	204	0.18	13.65	0.70	Violent or Angry	*Angry, abuse, anger, threatening*
T1.1.2	208	0.66	486	0.42	26.50	0.64	Time: Present; simultaneous	*Now, today, at the moment, currently*
T2-	89	0.28	213	0.19	10.23	0.60	Time: Ending	*Stop, ended up*
B2-	310	0.98	747	0.65	34.58	0.59	Disease	*Psychosis, symptoms, unwell, disorder, ill*
N4	209	0.66	520	0.45	20.07	0.54	Linear order	*Then, first, last, finally*
T2+	88	0.28	222	0.19	7.88	0.52	Time: Beginning	*Started, start, finally*
E6-	160	0.51	407	0.35	13.73	0.51	Worry	*Anxiety, worry, stress, worried*

^a^LL: Log Likelihood.

^b^Italicized text represents example words.

**Table 6:** Themes illustrating how relatives used the forum.

Theme	Title	Text
Time, including beginnings, endings, age and recency	How to get supported housing	“Just over a month ago my husband was diagnosed as having BD. Last Thursday after a very violent outburst with police involved and trip to hospital my eldest son who is 35 has been diagnosed with borderline personality disorder. He was off work last week with a physical problem, but today is hiding under the bedcovers having not slept with worrying.”
Negative emotions, including fear, depression, and anxiety	Starting a family	“I'm really struggling at the moment, and no one really to talk to, and even when I do they don't really seem to understand... It’s so exhausting keeping up with the switches and changes, the rejection and lack of empathy...”
Conflict and abuse, including anger, threats, and abuse	How to deal with verbal abuse and aggression	“I was wondering if anyone had any suggestions on how to handle abusive behaviour? My husband is hypomanic at the moment and this unfortunately involves aggressive and abusive behaviour—shouting, bullying, demeaning, berating, controlling...”
Illness and hospitalization, including psychosis and discharge	How to get supported housing	“Good morning all. I need some advice on how to get some help for my son. He is currently living in a general purpose housing association flat. He...has schizophrenia, with alcohol and gambling addictions. [...] His life is absolutely chaotic... [...] I have talked to his care coordinator but she seems to have no time to help us...Has anyone any advice?”
Death and suicide	How can we support him?	“He has a history of attempting suicide and we are so scared that we will lose him and that is his ultimate threat. His Dad and I are at the point that we can't support him at this level any more.”

## Discussion

### Principal Findings

This paper drew on data from a national trial examining the effectiveness of interactive digital support for relatives of people with psychosis or BD. Using detailed forum data, this paper explored who used the forum and why; how sociodemographic characteristics were associated with participation; the relationship among frequency, type of use, and outcomes; and how the forum was used.

In terms of who used the forum and why, it was found that there were no differences in sociodemographic variables among users, observers, and nonusers. Relatives who accessed the forum covered the whole age range; those aged ≥70 years did not include active forum participants but did include observers. There was a range of reasons behind participants’ first use of the forum. Typically, relatives’ first posts indicated a desire to connect with others in similar circumstances and share experiences rather than specific requests for advice or information.

It was found there were no associations between patterns of use and sociodemographic variables. Women and participants who had engaged with higher education were more likely to be users, but the differences were small. This contrasts with prior research on the use of digital resources in general and may be linked to the level of need for support in this group of carers [27].

Previous research has indicated that the 1% rule (90% of social media users observe but do not participate, 9% contribute in a limited way, and 1% contribute substantially [38,39]) applies to a number of digital social networks for mental health. In contrast to this, in this study, 59% (207/348) of the people accessed the forum and of those who did, 33% (67/207) posted. This level of engagement may reflect the lack of other support available to the relatives in this study. Levels of use were not significantly associated with relatives’ well-being at any of the assessment points in the study. This may reflect previous research indicating that observers can benefit from reading posts without posting themselves [40].

To determine how the forum was used, patterns of relative-initiated and REACT supporter–initiated posts were compared. Relatives engaged more strongly with domains linked to acute issues with their relatives and their own personal situation. These spanned across themes of time, negative emotions, conflict and abuse, and death and suicide. The details provided in posts of these types highlight the scale of the challenges experienced by relatives and the fact that they felt safe to share extensive and often painful personal details in this context. This is consistent with qualitative interviews with REACT participants, which highlighted the crucial importance of peer support through both REACT supporters and through sharing with other relatives [25].

### Comparisons With Prior Work

The reasons relatives joined the forum mainly consisted of a desire to connect with others and share their experiences, with relatives identifying the value of peer support and information sharing in their posts. This is consistent with the wider literature on mental health that indicates that people with lived experiences are often successful in promoting hope, empowerment, and social inclusion in peers by sharing personal experiences [41]. Relatives felt that they were able to offer detailed information about their experiences, possibly aided by the anonymity of the platform. Anonymity was confirmed to be important in qualitative interviews with the participants, published in the study by Lobban et al [25].

Previous research has suggested that older people do not use forums [42]; however, our findings suggest that older people do use forums, but they may be less active. Previous research has also suggested that digital resources might better serve people with higher levels of education [43], which is a finding that, this study supports, as people with higher levels of education tended to use the forums more.

Previous research on forum use by relatives has been extremely limited. Smith-Merry et al [22] conducted qualitative interviews with relatives and people living with psychosis from the Schizophrenia a National Emergency Australia forums. Consistent with this study, participants highlighted the importance of social connections, information, and practical advice, although it was not possible to identify the priorities of relatives. Terbeck and Chesterman [22] explored posts in 5 different forums by parents of children with suspected attention-deficit or hyperactivity disorder. Their content analysis indicated that parents typically received empathic and supportive responses to their initial posts, predominantly regarding dissatisfaction with professionals. This led the authors to suggest that such forums may decrease faith in health services and lead to “doctor shopping.” This was not the dominant pattern in the REACT forum data. Some participants did post about service limitations, but this was part of a range of topics that went beyond clinical care. Mazur and Mickle [23] explored web-based forum posts of parents of children with attention-deficit or hyperactivity disorder, BD, and depressive and anxiety disorders across 4 different forums. Content analysis indicated the importance of advice seeking and addressing feelings of helplessness across parents as well as concerns regarding verbal or physical conflict in relation to their child. Similar themes arose in this study, particularly around concerns regarding conflicts and abuse in relation to one’s relatives.

### Limitations

Although this paper provides important insights into forum use among relatives of people with serious mental illness, caution is needed when generalizing the results to the general population of relatives. This study was based on users of the REACT forum, all of whom were highly distressed and were taking part in the REACT trial. They may differ from those not experiencing high levels of distress or not actively involved in research and in seeking support because of their caring role. Furthermore, people from ethnic minority backgrounds were underrepresented in this study, as the overwhelming majority (331/348, 95%) of participants were from a White ethnic background. Future studies would benefit from a more diverse sample.

### Conclusions

Overall, this study indicates that, although only a proportion of users of digital support interventions for relatives engage actively with the forums, they are widely distributed across age and other sociodemographic groupings. Sociodemographic variables were not linked to levels of use. Relatives used the forums for information, support, and guidance and felt that they were able to offer detailed information about their experiences, possibly aided by the anonymity of the platform. Anonymity was confirmed as important in qualitative interviews with the participants, published in the study by Lobban et al [25]. Given that some common themes emerged, which may be useful for other forum user groups, development of good practice guidance across user groups is important for future studies to provide an understanding of forum use and the associated benefits and challenges at a larger scale. The topics raised highlight the extent of the burden carried by relatives and the potential value of easy-access, moderated, peer-supported forums in helping relatives to manage the challenges in their lives.

## Abbreviations

BD: bipolar disorder
GHQ: General Health Questionnaire
REACT: Relatives Education and Coping Toolkit
USAS: University Centre for Computer Corpus Research on Language Semantic Analysis System
